# Application of machine learning in predicting aggressive behaviors from hospitalized patients with schizophrenia

**DOI:** 10.3389/fpsyt.2023.1016586

**Published:** 2023-03-20

**Authors:** Nuo Cheng, Meihao Guo, Fang Yan, Zhengjun Guo, Jun Meng, Kui Ning, Yanping Zhang, Zitian Duan, Yong Han, Changhong Wang

**Affiliations:** ^1^Department of Clinical Medicine, Zhengzhou University, Zhengzhou, Henan, China; ^2^Department of Infection Prevention and Control, The Second Affiliated Hospital of Xinxiang Medical University, Xinxiang, Henan, China; ^3^Henan Mental Disease Prevention and Control Center, The Second Affiliated Hospital of Xinxiang Medical University, Xinxiang, Henan, China; ^4^Editorial Department of Journal of Clinical Psychosomatic Diseases, The Second Affiliated Hospital of Xinxiang Medical University, Xinxiang, Henan, China; ^5^Department of Medical Administration, The Second Affiliated Hospital of Xinxiang Medical University, Xinxiang, Henan, China; ^6^Department of Medicine, Zhengzhou University, Zhengzhou, Henan, China; ^7^The Seventh Psychiatric Department, The Second Affiliated Hospital of Xinxiang Medical University, Xinxiang, Henan, China; ^8^Henan Key Laboratory of Biological Psychiatry, The Second Affiliated Hospital of Xinxiang Medical University, Xinxiang, Henan, China; ^9^Department of Clinical Psychiatry, The Second Affiliated Hospital of Xinxiang Medical University, Xinxiang, Henan, China

**Keywords:** schizophrenia, predictive models, machine learning, aggressive behaviors, risk factors

## Abstract

**Objective:**

To establish a predictive model of aggressive behaviors from hospitalized patients with schizophrenia through applying multiple machine learning algorithms, to provide a reference for accurately predicting and preventing of the occurrence of aggressive behaviors.

**Methods:**

The cluster sampling method was used to select patients with schizophrenia who were hospitalized in our hospital from July 2019 to August 2021 as the survey objects, and they were divided into an aggressive behavior group (611 cases) and a non-aggressive behavior group (1,426 cases) according to whether they experienced obvious aggressive behaviors during hospitalization. Self-administered General Condition Questionnaire, Insight and Treatment Attitude Questionnaire (ITAQ), Family APGAR (Adaptation, Partnership, Growth, Affection, Resolve) Questionnaire (APGAR), Social Support Rating Scale Questionnaire (SSRS) and Family Burden Scale of Disease Questionnaire (FBS) were used for the survey. The Multi-layer Perceptron, Lasso, Support Vector Machine and Random Forest algorithms were used to build a predictive model for the occurrence of aggressive behaviors from hospitalized patients with schizophrenia and to evaluate its predictive effect. Nomogram was used to build a clinical application tool.

**Results:**

The area under the receiver operating characteristic curve (AUC) values of the Multi-Layer Perceptron, Lasso, Support Vector Machine, and Random Forest were 0.904 (95% CI: 0.877–0.926), 0.901 (95% CI: 0.874–0.923), 0.902 (95% CI: 0.876–0.924), and 0.955 (95% CI: 0.935–0.970), where the AUCs of the Random Forest and the remaining three models were statistically different (*p* < 0.0001), and the remaining three models were not statistically different in pair comparisons (*p* > 0.5).

**Conclusion:**

Machine learning models can fairly predict aggressive behaviors in hospitalized patients with schizophrenia, among which Random Forest has the best predictive effect and has some value in clinical application.

## Introduction

Schizophrenia, as a group of severe psychiatric disorders, has an unknown etiology and is mostly characterized by multiple impairments in perception, emotion, thinking, and behavior, as well as uncoordinated mental activity. Previous studies have shown that the most common type of psychiatric disorder in which aggressive behavior occurs or in which delinquency occurs is schizophrenia (1, 2). The prevalence of threatening and aggressive behavior is common in hospitalized schizophrenia patients, ranging from 15.3 to 53.2% (3). Aggressive behaviors refers to verbal or physical behavior with hostile intent, destroying objects or attacking others (4). Meta-analysis showed that 15.3–53.2% of hospitalized patients with schizophrenia in China had experienced aggressive behaviors during hospitalization, and the incidence of aggressive behaviors after the combination was 35.14% (3). The most frequent targets of patients’ aggressive behaviors were psychiatric medical staff, and a cross-sectional survey data from the MatchRN Psychiatry study found that almost 30% of nurses had been subjected to a serious assault in their professional lifetimes (5). The negative consequences of aggressive behaviors directly affect the safety and physical/mental health of patients themselves as well as others. And it increases the use of mandatory medical measures such as restraint and isolation, which raised the medical and family economic burden. Therefore, accurate assessment, risk warning and effective intervention of aggressive behaviors from hospitalized patients with schizophrenia have become the focus of psychiatric clinical work. Machine learning, which belongs to the branch of artificial intelligence, has been widely used in the medical field by constructing models through self-learning in big data and then making predictions on new data sets. Broadly speaking, machine learning is a computational technique that trains, learns, and gives solutions from input data sets (6). In psychiatric field, machine learning has been used by scholars to predict the response of patients with schizophrenia to repeated transcranial magnetic stimulation as well as suicide attempts of patients with schizophrenia, which works well in prediction (7, 8). Then a research from Gallos et al. (9) showed the ISOMAP and machine learning algorithms for the construction of embedded functional connectivity networks of anatomically separated brain regions from resting state fMRI data of patients with Schizophrenia, which also utilizes Random forest and Lasso for both diagnosing and finding biomarkers for the disease. Similarly, other studies from Gallos et al. (10, 11) tried to diagnose schizophrenia and find biomarkers for schizophrenia and tried to find ways to monitor treatments for Schizophrenia. A study that used machine learning algorithms to predict and find the influential factors of violence in male schizophrenia patients by Yu et al. (12). The aim of this study was to apply Multi-Layer Perceptron (MLP) (13), Lasso regression (14), Support Vector Machine (SVM) (15) and Random Forest (RF) (16) algorithms to predict aggressive behaviors of hospitalized patients with schizophrenia, to explore the application value of machine learning models in predicting aggressive behaviors of patients with schizophrenia.

## Materials and methods

### Study object

Patients with schizophrenia who were hospitalized in the Second Affiliated Hospital of Xinxiang Medical University from July 2019 to August 2021 were selected using the cluster sampling method. Inclusion criteria: (1) Meet the diagnostic criteria for schizophrenia in the International Statistical Classification of Diseases and Related Health Problem, Tenth Revision (ICD-10) (17); (2) no gender restriction, age ≥14 years old; (3) Education level of primary school or above, normal hearing and vision, able to understand and cooperate with the completion of the scale assessment; (4) previous outpatient or inpatient diagnosis for schizophrenia is needed, having taken antipsychotic drugs for 6 months or more. Exclusion criteria: (1) those with intellectual disability or combined organic brain disease; (2) those with severe physical illness or adverse drug reactions; (3) those with severe mental decline or excitement and agitation; (4) those with visual or auditory perception impairment; (5) pregnant or lactating female patients. The study was approved by the Ethics Committee of the Second Affiliated Hospital of Xinxiang Medical University, and the purpose and significance of the study were explained to the study subjects and their guardians, and written informed consent was obtained from the patients and their guardians.

### Research methods

#### Survey content

(1) General condition questionnaire: the questionnaire was developed by the subject members themselves based on previous studies (18–20) as well as the medical staff’s own experience, and was refined after a pre-survey to collect baseline information from the research objects. The questionnaire included: (1) basic information: age, gender, marital status, education, residence, occupation, caregiver, and family income; (2) disease information: duration of disease, times of hospitalizations, family history of schizophrenia, past attack history, and management style during hospitalization; (3) pre-admission status: medication adherence and subsequent visits. (2) Insight and Treatment Attitude Questionnaire (ITAQ) (21, 22): this scale was translated by Zhang Jing-hang etc. based on the 1989 version of McEvoy etc. The scale consists of 11 items, including knowledge of the disease and attitude toward treatment, and each item is rated on a 3-level scale from 0 to 2, with a total score of 0–22, in which higher total scores indicating better knowledge of the disease and better attitude toward treatment. The ITAQ had a retest reliability of 0.93 and a consistency reliability of 0.80. The stability of the ITAQ was good, and it was significantly correlated with the Positive Symptom Scale, the Negative Symptom Scale, and the Brief Psychiatric Scale, which showed that the ITAQ could accurately reflect patients’ conditions and had good validity in assessing patients’ insight. (3) The Family APGAR Questionnaire (23, 24): this scale was developed by Smikstein in 1978 to evaluate subjects’ subjective satisfaction with family functions. The scale contains five factors: Adaptation, Partnership, Growth, Affection, Resolve. The scale is rated on a 3-level Likert scale, with scores of 0 to 2 on terms of “rarely, sometimes, and often.” The total score is 0–10, and the higher the score is, the better the family function is; there are 3 levels, 7–10 for good family function, 4–6 for moderate family function, and 0–3 for poor family function. The scale has a retest reliability of 0.80–0.83. (4) Social Support Rating Scale (SSRS) (25, 26): It was developed in 1986 by Xiao Shui Shui etc. on the basis of reference to relevant international data, and the evaluation index includes 3 dimensions of subjective support, objective support and support utilization, with a total of 10 items. The scale can be summarized into 4 statistical indicators, with objective and subjective support factor scores ranging from 2 to 22 and 8 to 32, respectively; support utilization factor scores ranging from 3 to 12; and total scores ranging from 13 to 66, with higher scores resulting in more social support. The α coefficient of the total scale was 0.69, subjective support was 0.849, objective support was 0.825; support utilization was 0.833 which indicating a high reliability coefficient of internal consistency of each subscale. (5) Family Burden Scale of Disease (FBS) (27): a semidefinite scale developed by Pai and Kapur in 1981 for families of patients with mental disease to evaluate the burden of the disease on the family and its members. The scale includes 6 dimensions: family daily activities, financial burden, family recreational activities, physical health of family members, family relationships, and psychological health of family members, with a total of 24 items. Each item was rated on a 3-level scale from 0 to 2, with severe burden rated 2, moderate burden rated 1, and no burden rated 0. The higher the score, the heavier the burden on the family. The Cronbach’s α coefficient of the scale was 0.87–0.99. (6) Aggressive behaviors: The Modified Overt Aggression Scale (MOAS) (28), it was applied to evaluate patients’ aggressive behaviors before they were discharged from the hospital, and a weighted total score of 4 or more was used as the inclusion criterion for the “group with significant aggressive behavior.” The MOAS sort out four categories of aggressive behaviors, all of which were rated on 5-levels scale from 0 to 4, and weighted scores were set for different aggressive behaviors. The scorer reliability was tested, and the consistency among multiple raters was good, the Intraclass Correlation Coefficient (ICC) of the scale was 0.84 (*p* < 0.01). Taking “whether obvious aggressive behaviors occurs” as the dependent variable, and “factors influencing aggressive behaviors” as the independent variable, all the types and codes of all variables are shown in Table 1.

**Table 1:** List of hyperparameters for each model.

Models	Hyperparameters
RF	Max_features = 0.17944531866616362 min_samples_split = 13 n_estimators = 245 (number of trees)
SVM	Kernal = ‘rbf’ expC = 18.598047792385053 expGamma = −27.730357792272096
MLP	Hidden_layer_sizes = (80,96) alpha = −251.94363317811685 activation = ‘relu’
Lasso	Alpha = −45.30976305646013

The other hyperparameters adopted the default settings in the “sklearn” package of python.

### Survey method

All surveys were completed by 6 psychiatric attending physicians and 6 psychiatric nurse practitioners from our hospital who were well trained for this subject in purpose of having a consistent data criteria. (1) The survey of baseline information was completed by psychiatric nurses within 3 days after the patients were enrolled. The out-of-hospital medication adherence can be defined as the compliance level to which a patient’s medication-taking matches the prescriber. Good adherence refers to the patient’s long-term adherence to medication in full compliance with the doctor’s prescription; moderate adherence refers to the fact that the patient cannot take the medicine exactly as prescribed by the doctor, including taking less or more, not on time, or missing sometimes; poor adherence refers to the patient not taking medication as prescribed frequently, often not taking medication, or stopping taking medication. The subsequent vists was evaluated by: the patient’s family was asked about the subsequent vists in the last 6 months, the specific evaluation method is shown in Table 1. (2) The ITAQ, APGAR, SSRS and FBS surveys were conducted by the psychiatrist within 3 days after the patient’s enrollment. The survey will use unified guidelines. The assessor can read each question to the patients and guardians. Where they do not understand can be explained in details. (3) After the questionnaire was collected, invalid questionnaires with missing items or inconsistencies were excluded. (4) The MOAS scale was used by the psychiatric nurses to investigate whether the patients had any aggressive behaviors during the hospitalization before discharge from the hospital.

### Machine learning methods

In this study, several machine learning algorithms are selected for modeling, and different models have their own characteristics. The Multi-Layer Perceptron consists of an input layer, an output layer, and a “hidden” layer between them, and the connections between neurons in each layer are given weights, which are continuously adjusted during the generation process to train the learning algorithm and minimize the prediction error, due to the large number of parameters. The algorithm is prone to overfitting and gradient exploding (29). The Lasso adds L2 regularization term based on the general linear model to compress the coefficients with small absolute values to zero, so as to achieve the purpose of having variables selected and parameters estimated at the same time, which overcomes the selection methods limitations of stepwise regression variables while retaining the excellent properties of subset selection and ridge regression (30). The Random Forest is an integrated algorithm that consisted of multiple decision trees (31) with a very high accuracy, a decent interpretability of the results and the ability to evaluate the importance of each feature in the classification. Gini importance algorithm, which measures the importance of each feature by calculating the mean of impurity decrease across all trees in the forest, was utilized for rank the relative importance of features. Support Vector Machine algorithms are theoretically based on nonlinear mapping, where features can be mapped to a high-dimensional space by kernel functions and then find a “hyperplane” that can be used for classification (8). Support Vector Machines can avoid “Curse of Dimensionality” to a certain extent. And robustness and generalization is good in performance, but it’s difficult for SVM to be used for training large-scale samples and it is sensitive to parameters and kernel functions. In summary, each of the four algorithms has its own characteristics and advantages, and the Random Forest achieved the best fitting in this study.

### Model building and validation

**Figure 1:**
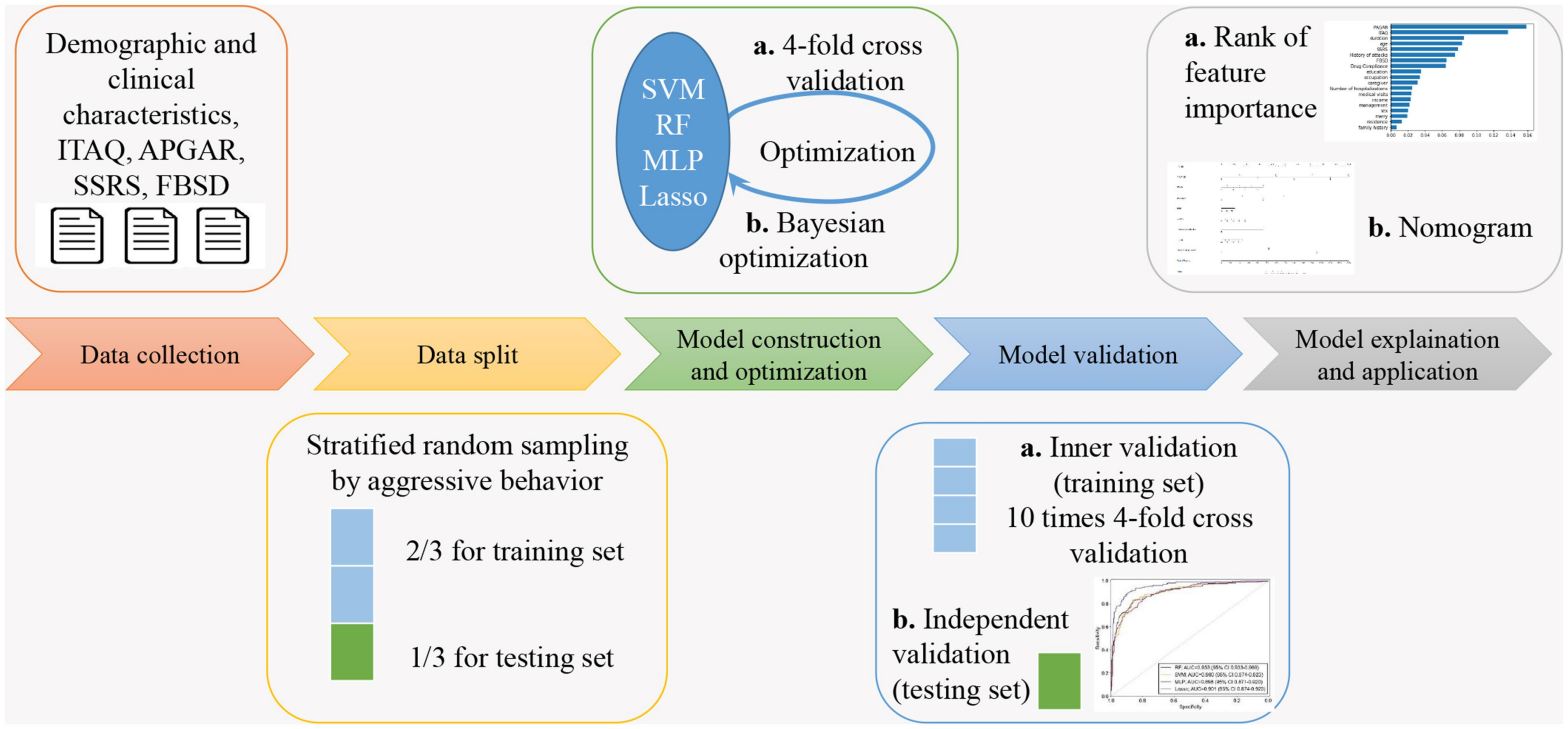
The flowchart of the methodology.

In this subject, the Python libraries “numpy,” “pandas” and “scikit-learn” were used for data processing as well as machine learning model building and validation. The samples were randomly divided into training set (70%) and testing set (30%). The training set was used for model training and hyperparameter optimization. The Bayesian optimization (32) was used in the training phase to obtain the best hyperparameters for the model, while 4-fold cross-validation was used to ensure the stability of the selected hyperparameters. After the optimal hyperparameters were obtained, 10 times 4-fold cross validation conducted on the training set for inner validation, then the model was retrained on all training set data, and then the performance of the model is finally evaluated using the testing set. The evaluation indicators include accuracy, sensitivity, specificity, and area under the curve (AUC) of the receiver operating characteristic (ROC) curve. The optimal hyperparameters for each machine learning model are shown in Table 1, and the ROC curves of the models were statistically compared using the Delong test (33). The flowchart that sketches the methodology of this study is shown in Figure 1.

### Statistical analysis

Data entry had been double-checked with Epidata 3.0 by different researchers. SPSS23.0 was used for data statistical description and analysis. Count data were described by the number of cases and percentages, and the *χ*^2^ test was used for comparison; measurement data conforming to normal distribution were expressed by x ± s and compared by *t*-test; rank data were tested by rank sum test, and *p* < 0.05 was considered statistically significant difference.

## Results

### Basic information

A total of 2,184 patients with schizophrenia were enrolled in this study, 2,184 questionnaires were distributed, 2064 valid questionnaires were collected, and the effective collection efficiency of questionnaires was 94.51%. During the hospitalization period, 27 cases withdrew from the study for reasons such as automatic discharge from hospital or developed combined somatic diseases. A total of 2037 patients were included in the analysis, of which 611 cases (611/2037, 30.00%) showed obvious aggressive behaviors and 1,426 cases (1426/2037, 70.00%) did not show obvious aggressive behaviors. The basic profiles of the patients in both groups are shown in Table 2.

**Table 2:** Comparison of basic conditions between the two groups (num = 2037).

Item	Aggressive behavior group (*n* = 611, %)	Non-aggressive behavior group (*n* = 1,426, %)	Statistical value	*p-*value
Gender [number (%)]			30.828^(1)^	<0.001
Male	401 (65.63)	746 (52.31)		
Female	210 (34.37)	680 (47.69)		
Age (years, x̅ ±S)	35.37 ± 10.29	35.66 ± 10.62	−0.573^(2)^	0.566
Education level [number (%)]			−0.361^(3)^	0.718
Primary school	15 (2.45)	41 (2.88)		
Junior middle school	54 (8.84)	116 (8.13)		
High school/secondary technical school	255 (41.73)	583 (40.88)		
Junior college	226 (36.99)	545 (38.22)		
Bachelor’s degree and above	61 (9.98)	141 (9.89)		
Marital status [number (%)]			0.387^(1)^	0.943
Unmarried	213 (34.86)	486 (34.08)		
Married	360 (58.92)	857 (60.10)		
Divorced	32 (5.24)	68 (4.77)		
Widowed	6 (0.98)	15 (1.05)		
Occupation [number (%)]			0.485^(1)^	0.975
Student	72 (11.78)	163 (11.43)		
Farmer	313 (51.23)	745 (52.24)		
Laborer	133 (21.77)	303 (21.25)		
Cadre	55 (9.00)	120 (8.42)		
Other	38 (6.22)	95 (6.66)		
Monthly family income [number (%)]			−0.034^(3)^	0.973
<5,000 RMB	168 (27.50)	403 (28.26)		
5,000 ~ 10,000 RMB	292 (47.49)	661 (46.35)		
>10,000 RMB	151 (24.71)	362 (25.39)		
Residence [number (%)]			0.000^(1)^	0.985
Urban	166 (27.17)	388 (27.21)		
Rural	445 (72.83)	1,038 (72.79)		
Caregivers [number (%)]			21.297^(1)^	<0.001
Parents	245 (40.10)	602 (42.22)		
Spouse	292 (47.79)	737 (51.68)		
Siblings	55 (9.00)	65 (4.56)		
Other	19 (3.11)	22 (1.54)		
Duration of disease [number (%)]			−3.941^(3)^	<0.001
<2 years	148 (24.22)	410 (28.75)		
2 ~ 10 years	187 (30.61)	457 (32.05)		
>10 years<20 years	188 (30.77)	491 (34.43)		
20 years and above	88 (14.40)	68 (4.77)		
Family history of schizophrenia [number (%)]			0.007^(1)^	0.932
No	32 (5.24)	76 (5.33)		
Yes	579 (94.76)	1,350 (94.67)		
History of previous attacks [number (%)]			261.794^(1)^	<0.001
No	152 (24.88)	912 (63.96)		
Yes	459 (75.12)	514 (36.04)		
Times of hospitalizations [number (%)]			−0.876^(3)^	0.381
1time	69 (11.29)	127 (8.91)		
2 ~ 3 times	383 (62.68)	923 (64.73)		
4 times or more	159 (26.02)	376 (26.37)		
Management method during hospitalization [Number (%)]			67.957^(1)^	<0.001
Closed	523 (85.60)	969 (67.95)		
Open	88 (14.40)	457 (32.05)		
Medication adherence [number (%)]			141.463^(1)^	<0.001
Poor	355 (58.10)	430 (30.15)		
Medium	226 (36.99)	895 (62.76)		
Good	30 (4.91)	101 (7.08)		
Frequency of subsequent visits [number (%)]			60.614^(1)^	<0.001
1time in 4 ~ 5 months	371 (60.72)	598 (41.94)		
1 time in 2 ~ 3 months	225 (36.82)	771 (54.07)		
1 time in 1 month	15 (2.45)	57 (4.00)		
ITAQ (points, x̅ ±S)	5.73 ± 1.43	6.22 ± 1.44	−7.078^(2)^	<0.001
APGAR (points, x̅ ±S)	4.55 ± 1.58	5.88 ± 1.12	−21.335^(2)^	<0.001
SSRS (points, x̅ ±S)	23.92 ± 3.61	24.26 ± 2.43	−2.482^(2)^	0.013
FBS (points, x̅ ±S)	22.49 ± 3.37	22.26 ± 2.79	1.618^(2)^	0.106

(1) is the *χ*^2^ test; (2) is the *t* test; (3) is the rank sum test.

### Performance of machine learning models

**Figure 2:**
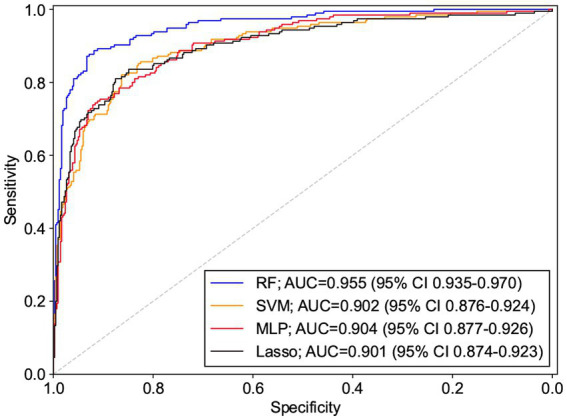
ROC and AUC values of different machine learning models on the testing set.

All 19 variables are included in the four machine learning algorithms for model fitting, and the included variables are shown in Table 3. The Random Forest was found to have the best prediction in the testing set, with a statistically different AUC from the remaining three models (*p* < 0.0001), and the remaining three models were not statistically different in pair comparisons (*p* > 0.5). The AUC predicted by RF was 0.955; the AUC predicted by SVM was 0.902; the AUC predicted by MLP was 0.904; and the AUC predicted by Lasso was 0.901, as shown Table 4. Figure 2 shows the ROC curves and AUC values of 4 different machine learning models on the testing set. The inner validation result shown in Table 5. To explore the effect of data imbalance on the model fitting effect, we randomly selected 611 cases of aggressive patients with the same number of non-aggressive patients, created a new dataset (balanced dataset), and repeated the above modeling evaluation process on the new dataset. The results showed that the performance of the models fitted to the balanced and unbalanced datasets was comparable, and the models fitted on the balanced dataset were slightly inferior to those fitted on the unbalanced dataset (Table 5).

**Table 3:** Names, types and codes of variables.

Variable name	Variable type	Variable code	Code method
Gender	Categorical variables	X_1_	1 = Male 2 = Female
Age	Continuous type variable	X_2_	(year)
Education level	Categorical variables	X_3_	1 = primary school 2 = Junior high school 3 = High school/Secondary school 4 = Junior College 5 = Bachelor and above
Marital Status	Categorical variables	X_4_	1 = unmarried 2 = married 3 = divorced 4 = widowed
Occupation	Categorical variables	X_5_	1 = Student 2 = Farmer 3 = Laborer 4 = Cadre 5 = Other
Monthly family income	Categorical variables	X_6_	1 = <5000RMB 2 = 5,000 to 10000RMB 3= > 10000RMB
Place of residence	Categorical variables	X_7_	1 = Urban 2 = Rural
Caregiver	Categorical variables	X_8_	1 = Parents 2 = Spouse 3 = Siblings 4 = Other
Duration of Disease	Categorical variables	X_9_	1 = <2 2 = 2 to 10 3= > 10 < 20 years 4= > 20 (years)
Family History of schizophrenia	Categorical variables	X_10_	1 = No 2 = Yes
History of previous attacks	Categorical variables	X_11_	1 = No 2 = Yes
Times of hospitalizations	Categorical variables	X_12_	1 = 1 time 2 = 2 to 3 times 3 = 4 times and more
Management style during hospitalization	Categorical variables	X_13_	1 = closed 2 = open
Medication adherence	Categorical variables	X_14_	1 = poor 2 = moderate 3 = good
Frequency of subsequent visits	Categorical variables	X_15_	1 = 4–5 months 2 = 2–3 months 3 = 1 month
ITAQ scores	Continuous variables	X_16_	(points)
APGAR scores	Continuous variables	X_17_	(points)
SSRS scores	Continuous variables	X_18_	(points)
FBS scores	Continuous variables	X_19_	(points)

**Table 4:** Performance of different machine learning models on the testing set.

Model	AUC (95%CI)	Accuracy	Sensitivity	Specificity
RF	0.955 (0.935–0.970)	0.889	0.892	0.887
SVM	0.902 (0.876–0.924)	0.827	0.949	0.770
MLP	0.904 (0.877–0.926)	0.866	0.908	0.847
Lasso	0.901 (0.874–0.923)	0.866	0.908	0.847

95%CI, 95% confidence interval.

**Table 5:** The AUC of each model in training sets of original dataset and balanced dataset by 10 times 4-fold cross validation.

Model	AUC (95%CI)	Accuracy	Sensitivity	Specificity
**Original dataset**				
RF	0.949 (0.938–0.960)	0.891	0.621	0.975
SVM	0.900 (0.884–0.916)	0.875	0.634	0.949
MLP	0.888 (0.872–0.904)	0.861	0.609	0.939
Lasso	0.884 (0.867–0.901)	0.868	0.617	0.945
**Balanced dataset**				
RF	0.933 (0.916–0.950)	0.855	0.860	0.850
SVM	0.898 (0.877–0.919)	0.829	0.797	0.861
MLP	0.886 (0.865–0.907)	0.813	0.789	0.836
Lasso	0.884 (0.863–0.905)	0.811	0.800	0.823

95%CI, 95% confidence interval.

### Rank of feature importance

**Figure 3:**
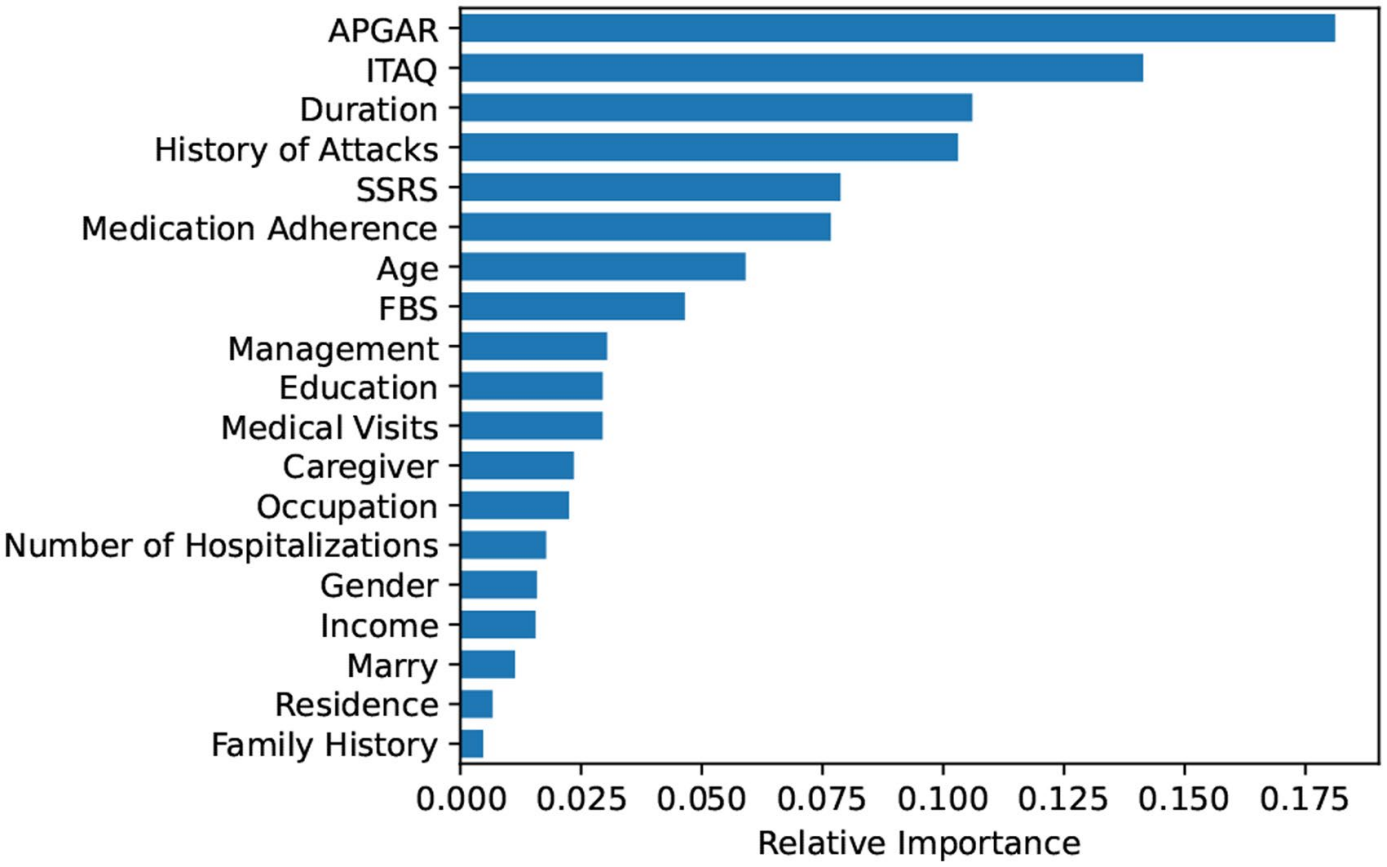
Rank of feature importance obtained by random forest.

**Figure 4:**
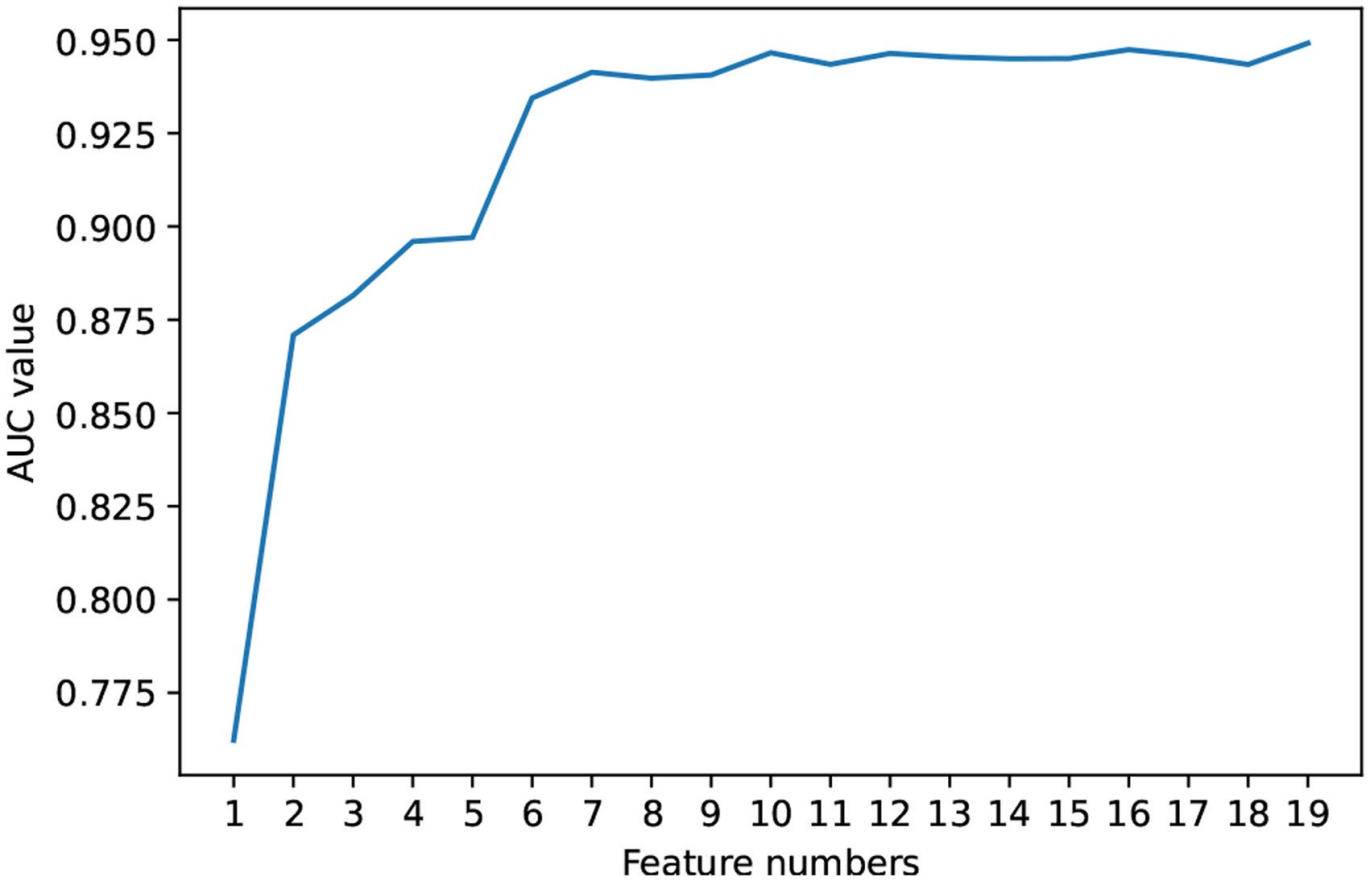
AUC values of random forest model versus number of top-ranked features.

Based on the Random Forest model, this study ranked the importance of the features in predictive value. The results are shown in Figure 3. Further, we included the ranked features in order of feature importance for modeling (Figure 4), and the results showed that the model performance was maintained at a stable plateau to some extent after the inclusion of the top 8 features (AUC = 0.9397). In addition, the model performs best when all features were included (AUC = 0.9491). The top 8 features were APGAR, ITAQ, Duration, History of Attacks, SSRS, Medication Adherence, Age, FBS.

### Prediction tools of constructing a nomogram

**Figure 5:**
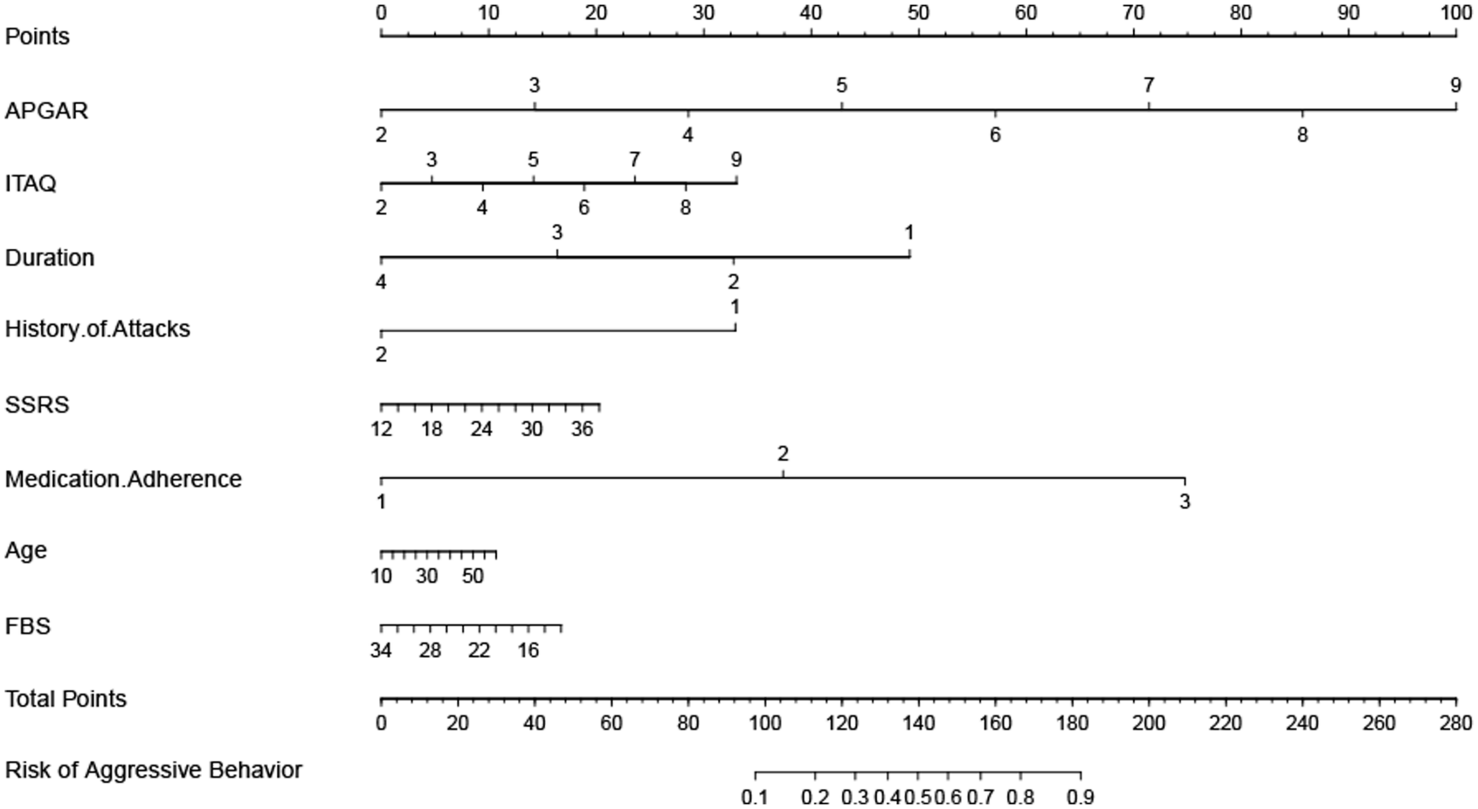
Nomogram based on the top 8 significant features (variables) filtered by Random Forest.

To facilitate clinical application, a nomogram was drawn based on the top eight significant variables obtained from the Random Forest model, as shown Figure 5. According to the scaleplate above the nomogram, the individual score of each risk factor is obtained, and all risk factors are added to obtain the total scores, and the total scores shows the probability of aggressive behaviors during hospitalization of each patient.

## Discussion

Aggressive behaviors in patients with schizophrenia can be very harmful to their family members, the patients themselves, and health care professionals. Currently, the prediction of aggressive behaviors of patients with schizophrenia in China mainly uses MOAS etc., and many other existing structured clinical risk assessment tools, which are very time-consuming (28). Current research on aggressive behaviors of patients with schizophrenia has mainly focused on analyzing its influencing factors using logistic regression. Wang et al. in Toronto reported the outcomes of a binary logistic regression model with six machine learning algorithms used in predicting violence status in 275 patients with schizophrenia, using various demographic, clinical, and sociocultural predictor variables. The study showed that the random forest model performed marginally better than other algorithms (34). Yu et al. in Hefei used eight machine learning algorithms to predict violent behavior in 397 male patients with schizophrenia. The Neural Net had better prediction ability than that of other algorithms (12). While in another study from Yu et al. with smaller samples of 57 male patients with schizophrenia, the Support Vector Machine performed the best (35). Sonnweber et al. in Zurich used files of 370 offender patients diagnosed with schizophrenia spectrum disorders, Gradient Boosting was identified as the best performing algorithm (36). Kirchebner et al. Studied the same samples of 370 offender patients in Zurich with a conclusion of Boosted Classification Trees as best suited (37). Guo et al. In Shenzhen examined 74 male participants with a hybrid machine learning model (LASSO regression and SVM), with an AUC of 0.95 (38). Please check the detailed performance of these studies in Table 6. These studies suggest that with further work, classification algorithms may have the ability to supplement diagnostic decisions to improve the treatment and well-being of patients with schizophrenia. In this study, multiple machine learning classification algorithms were used to predict aggressive behaviors of patients with schizophrenia based on multidimensional indicators such as demographic, clinical and social etc. The Random Forest algorithm showed value in prediction, and this study performed feature importance ranking to increase the interpretability of the model, and plotted a nomogram of tools for clinical application, highlighting the value for clinical application of this study.

**Table 6:** Studies using machine learning models for predicting risk of aggression and/or violence.

References	Year study published	Region	Male/female	No. of aggressive patients	Best performed algorithm	AUC	Accuracy/balanced accuracy	Sensitivity	Specificity
Wang et al. (34)	2020	Toronto, Canada	197/78	103	Random forest algorithm	0.63 ± 0.005	0.62 ± 0.004	0.32 ± 0.008	0.80 ± 0.004
Yu et al. (12)	2022	Hefei, China	397/0	146	Neural net	0.6673 (0.5599–0.7748)	0.6416	0.4444	0.8387
Yu et al. (35)	2022	Hefei, China	57/0	30	Support vector machine	0.8410 (0.6826–0.9995)	0.8231	0.8	0.8462
Sonnweber et al. (36)	2021	Zurich, Switzerland	338/31	294	Gradient boosting	0.8026	70.21%	68.22%	72.20%
Kirchebner et al. (37)	2020	Zurich, Switzerland	338/31	294	Boosted classification trees	0.83	76.40%	80.49%	71.19%
Gou et al. (38)	2021	Shenzhen, China	74/0	42	Hybrid machine learning (the LASSO regression and SVM)	0.95	90.67%	90.91%	90.48%

The top 8 features in Random Forest regarding prediction of aggressive behaviors from hospitalized patients with schizophrenia were: APGAR, ITAQ, Duration, History of Attacks, SSRS, Medication Adherence, Age, FBS. Previous studies have shown that family care of patients with schizophrenia is positively correlated with their mental health (39); some cognitive impairments appear to have their place in the genesis, progression and maintenance of violent acts of individuals with schizophrenia (40). cognitive impairment is more severe in patients with schizophrenia with a longer course of the illness (41, 42); a history of previous aggressive behaviors or being a victim of violent attacks can be a risk factor for increases of aggressive behaviors (43); poor social support (such as poor communication with family members or difficulties in getting along), treatment and medication poor adherence, and a heavy family burden that leaves patients with no family care can increase the risk of aggressive or even violent behaviors from patients (44, 45). Combined with the results of this study, it suggests that not only should patients with schizophrenia be treated in a standardized manner in the clinic, but also their family members should be included in the treatment activities. Family education and training can be carried out to enhance the family members’ acceptance to patients, to eliminate the negative attitudes such as discrimination against patients within the family, to help patients to recover faster in a good family atmosphere, to improve their insight and attitude towards treatment.

Nomogram is a statistical model for individualized predictive analysis of clinical events, which can provide better individualized predictive risk assessment in an intuitive and visual way (46, 47). In this study, a visual nomogram model was established based on the integration of 8 important variables screened out by the Random Forest. The calibration curve and ROC curve analysis showed that this model had good predictive calibration and discrimination. This model can be used by health care professionals to predict the probability of aggressive behaviors in hospitalized patients with schizophrenia based on the summation of the scores of each risk factor, to identify high-risk groups, to provide early warning of risks, to develop effective treatment and intervention measures, to prevent or mitigate the adverse consequences of aggressive behaviors, to improve safety management in psychiatric departments, and to create a healthy environment for patients and health care professionals.

This study constructed a predictive model of aggressive behaviors by investigating the baseline data, disease conditions, and scale assessments of 2,037 patients with schizophrenia, which is superior to traditional scale predictive methods. It can accurately reflect the influential factors leading to the aggressive behaviors of patients with schizophrenia, providing reliable data support for psychiatric clinical prediction and prevention of aggressive behaviors which can reduce the losses. The deficiencies of this study: first, this study aimed to build a prediction model, data collection had to be completed at the initial stage of patient admission, so the impact of the patient’s disease stage cannot be studied; second, this study only analyzed general demographic information and disease-related factors of patients with schizophrenia, but did not collect predisposing factors and biological indicators for the occurrence of aggressive behaviors. More variables need to be further explored. Subsequent studies can further improve the above deficiencies to systematically study the biological, psychological, and social factors of patients with schizophrenia in purpose of predicting aggressive behaviors more accurately and ensuring the safety in psychiatric clinical practices.

In summary, machine learning algorithms can be used for risk prediction of aggressive behaviors from patients with schizophrenia. This study has some value in clinical application and it is conducive to the clinical development of differentiated management and precise nursing against aggressive behaviors from patients with schizophrenia of different types.

## Data availability statement

The original contributions presented in the study are included in the article/Supplementary material, further inquiries can be directed to the corresponding authors.

## Ethics statement

The studies involving human participants were reviewed and approved by Ethics Committee of the Second Affiliated Hospital of Xinxiang Medical University. Written informed consent to participate in this study was provided by the participants’ legal guardian/next of kin.

## Author contributions

CW contributed to conception and design of the study. MG, FY, and ZG organized the database. YH performed the statistical analysis. NC and MG wrote the first draft of the manuscript. JM, YZ, ZD, and KN wrote sections of the manuscript. JM edited the manuscript. CW contributed to project administration. All authors contributed to the article and approved the submitted version.

## Funding

This study was supported by Henan Provincial Clinical Medical Research Center for Psychiatric and Psychological Disorders (2019-zxkfkt-009), Central Plains Talent Programme (Yucai Series), “the Open Project of Henan Key Lab of Biological Psychiatry [ZDSYS2021005]”, “the Joint Co-construction Project of Henan Medical Science and Technology Research Plan [LHGJ20220637]”, and “Construction of “Internet +” continuous nursing service model and its application in stable stage patients with severe mental disorders in community [RKX202202038].

## Conflict of interest

The authors declare that the research was conducted in the absence of any commercial or financial relationships that could be construed as a potential conflict of interest.

## Publisher’s note

All claims expressed in this article are solely those of the authors and do not necessarily represent those of their affiliated organizations, or those of the publisher, the editors and the reviewers. Any product that may be evaluated in this article, or claim that may be made by its manufacturer, is not guaranteed or endorsed by the publisher.

## References

[1] Krakowski MI Czobor P. Distinctive profiles of traits predisposing to violence in schizophrenia and in the general population. Schizophr Res. (2018) 202:267–73. doi: 10.1016/j.schres.2018.07.008, PMID: 30021703

[2] Rund BR. A review of factors associated with severe violence in schizophrenia. Nord J Psychiatry. (2018) 72:561–71. doi: 10.1080/08039488.2018.1497199, PMID: 30099913

[3] Zhou JS Zhong BL Xiang YT Chen Q Cao XL Correll CU. Prevalence of aggression in hospitalized patients with schizophrenia in China: a meta-analysis. Asia Pac Psychiatry. (2016) 8:60–9. doi: 10.1111/appy.12209, PMID: 26346165

[4] Anderson CA Bushman BJ. Human aggression. Annu Rev Psychol. (2002) 53:27–51. doi: 10.1146/annurev.psych.53.100901.135231 11752478

[5] Schlup N Gehri B Simon M. Prevalence and severity of verbal, physical, and sexual inpatient violence against nurses in Swiss psychiatric hospitals and associated nurse-related characteristics: cross-sectional multicentre study. Int J Ment Health Nurs. (2021) 30:1550–63. doi: 10.1111/inm.12905, PMID: 34196092 PMC8596810

[6] Dwyer DB Falkai P Koutsouleris N. Machine learning approaches for clinical psychology and psychiatry. Annu Rev Clin Psychol. (2018) 14:91–118. doi: 10.1146/annurev-clinpsy-032816-045037 29401044

[7] Koutsouleris N Wobrock T Guse B Langguth B Landgrebe M Eichhammer P. Predicting response to repetitive transcranial magnetic stimulation in patients with schizophrenia using structural magnetic resonance imaging: a multisite machine learning analysis. Schizophr Bull. (2018) 44:1021–34. doi: 10.1093/schbul/sbx114, PMID: 28981875 PMC6101524

[8] Hettige NC Nguyen TB Yuan C Rajakulendran T Baddour J Bhagwat N. Classification of suicide attempters in schizophrenia using sociocultural and clinical features: a machine learning approach. Gen Hosp Psychiatry. (2017) 47:20–8. doi: 10.1016/j.genhosppsych.2017.03.001, PMID: 28807134

[9] Gallos IK Gkiatis K Matsopoulos GK Siettos C. Isomap and machine learning algorithms for the construction of embedded functional connectivity networks of anatomically separated brain regions from resting state Fmri data of patients with schizophrenia. AIMS Neurosci. (2021) 8:295–321. doi: 10.3934/Neuroscience.2021016, PMID: 33709030 PMC7940114

[10] Gallos IK Galaris E Siettos CI. Construction of embedded Fmri resting-state functional connectivity networks using manifold learning. Cogn Neurodyn. (2021) 15:585–608. doi: 10.1007/s11571-020-09645-y, PMID: 34367362 PMC8286923

[11] Gallos IK Mantonakis L Spilioti E Kattoulas E Savvidou E Anyfandi E. The relation of integrated psychological therapy to resting state functional brain connectivity networks in patients with schizophrenia. Psychiatry Res. (2021) 306:114270. doi: 10.1016/j.psychres.2021.114270, PMID: 34775295

[12] Yu T Zhang X Liu X Xu C Deng C. The prediction and influential factors of violence in male schizophrenia patients with machine learning algorithms. Front Psych. (2022) 13:799899. doi: 10.3389/fpsyt.2022.799899, PMID: 35360130 PMC8962616

[13] Isler Y. Discrimination of systolic and diastolic dysfunctions using multi-layer perceptron in heart rate variability analysis. Comput Biol Med. (2016) 76:113–9. doi: 10.1016/j.compbiomed.2016.06.029, PMID: 27424172

[14] Dai P Chang W Xin Z Cheng H Ouyang W Luo A. Retrospective study on the influencing factors and prediction of hospitalization expenses for chronic renal failure in China based on random Forest and Lasso regression. Front Public Health. (2021) 9:678276. doi: 10.3389/fpubh.2021.678276, PMID: 34211956 PMC8239170

[15] Gul A Perperoglou A Khan Z Mahmoud O Miftahuddin M Adler W. Ensemble of a Subset of Knn classifiers. Adv Data Anal Classif. (2018) 12:827–40. doi: 10.1007/s11634-015-0227-5, PMID: 30931011 PMC6404785

[16] Cherkassky V. The nature of statistical learning theory~. IEEE Trans Neural Netw. (1997) 8:1564. doi: 10.1109/tnn.1997.641482 18255760

[17] Brämer GR. International statistical classification of diseases and related health problems. Tenth Revision World Health Stat Q. (1988) 41:32–6. 3376487

[18] Sariaslan A Arseneault L Larsson H Lichtenstein P Fazel S. Risk of subjection to violence and perpetration of violence in persons with psychiatric disorders in Sweden. JAMA Psychiat. (2020) 77:359–67. doi: 10.1001/jamapsychiatry.2019.4275, PMID: 31940015 PMC6990843

[19] Witt K van Dorn R Fazel S. Risk factors for violence in psychosis: systematic review and meta-regression analysis of 110 studies. PLoS One. (2013) 8:e55942. doi: 10.1371/journal.pone.0055942, PMID: 23418482 PMC3572179

[20] Appelbaum PS. In search of a new paradigm for research on violence and schizophrenia. Am J Psychiatry. (2019) 176:677–9. doi: 10.1176/appi.ajp.2019.19070678, PMID: 31474122

[21] Zhang JX Li XB Wong Z Liu JT Liu XC Yuan W. Clinical trial of insight and treatment attitude questionnaire. Shandong Arch. Psychiatry. (1994) 04:10–3.

[22] McEvoy JP Apperson LJ Appelbaum PS Ortlip P Brecosky J Hammill K. Insight in schizophrenia. Its relationship to acute psychopathology. J Nerv Ment Dis. (1989) 177:43–7. doi: 10.1097/00005053-198901000-00007, PMID: 2562850

[23] Smilkstein G Ashworth C Montano D. Validity and reliability of the family Apgar as a test of family function. J Fam Pract. (1982) 15:303–11. 7097168

[24] Lu JH Lang YL Guan HJ Lv YM Zhao C. Influences of family concern and self-efficacy on acceptance of disability of post-stroke disabled patients. Chin J Gerontol. (2020) 06:1324–8. doi: 10.3969/j.issn.1005-9202.2020.06.063

[25] Zhang MY He YL. Hand Book of Rating Scales in Psychiatry: Changsha, Changsha, Hunan, China: Hunan Science & Technology Press (2016). 384–387 p.

[26] Xiao SY. Theoretical basis and research application of social support rating scale. J Clin Psychiatry. (1994) 02:98–100.

[27] Pai S Kapur RL. Impact of treatment intervention on the relationship between dimensions of clinical psychopathology, social dysfunction and burden on the family of psychiatric patients. Psychol Med. (1982) 12:651–8. doi: 10.1017/s0033291700055756, PMID: 7134321

[28] He JF Hong W Shao Y Han HQ Xie B. Application of Moas for evaluating of violence risk in the inpatients with mental disorders. Fa Yi Xue Za Zhi. (2017) 33:28–31. doi: 10.3969/j.issn.1004-5619.2017.01.007, PMID: 29231005

[29] Yi K Ba WJ Tang M Liu JS Wang Q Sun TT. Multilayer perceptron model in predicting in filtration degree of pure ground glass opacity lung adenocarcinoma. Chin J Med Imaging Technol. (2020) 11:1652–6. doi: 10.13929/j.issn.1003-3289.2020.11.014

[30] Han YF Qin WF Chen W Li BH Teng BG Fang Y. Influencing factors on Elder’s preference for supporting: application of an adaptive Lasso logistic model. Chin. J. Health Stat. (2017) 1:18–22.

[31] Cutler A Stevens JR. Random forests for microarrays. Methods Enzymol. (2006) 411:422–32. doi: 10.1016/s0076-6879(06)11023-x 16939804

[32] Shields BJ Stevens J Li J Parasram M Damani F Alvarado JIM. Bayesian reaction optimization as a tool for chemical synthesis. Nature. (2021) 590:89–96. doi: 10.1038/s41586-021-03213-y, PMID: 33536653

[33] Demler OV Pencina MJ D'Agostino RB Sr. Misuse of Delong test to compare Aucs for nested models. Stat Med. (2012) 31:2577–87. doi: 10.1002/sim.5328, PMID: 22415937 PMC3684152

[34] Wang KZ Bani-Fatemi A Adanty C Harripaul R Griffiths J Kolla N. Prediction of physical violence in schizophrenia with machine learning algorithms. Psychiatry Res. (2020) 289:112960. doi: 10.1016/j.psychres.2020.112960, PMID: 32361562

[35] Yu T Pei W Xu C Zhang X Deng C. Prediction of violence in male schizophrenia using Smri, based on machine learning algorithms. BMC Psychiatry. (2022) 22:676. doi: 10.1186/s12888-022-04331-1, PMID: 36320010 PMC9628088

[36] Sonnweber M Lau S Kirchebner J. Violent and non-violent offending in patients with schizophrenia: exploring influences and differences via machine learning. Compr Psychiatry. (2021) 107:152238. doi: 10.1016/j.comppsych.2021.152238, PMID: 33721584

[37] Kirchebner J Sonnweber M Nater UM Gunther M Lau S. Stress, schizophrenia, and violence: a machine learning approach. J Interpers Violence. (2022) 37:602–22. doi: 10.1177/0886260520913641, PMID: 32306866

[38] Gou N Xiang Y Zhou J Zhang S Zhong S Lu J. Identification of violent patients with schizophrenia using a hybrid machine learning approach at the individual level. Psychiatry Res. (2021) 306:114294. doi: 10.1016/j.psychres.2021.114294, PMID: 34823086

[39] Zimmer M Duncan AV Laitano D Ferreira EE Belmonte-de-Abreu P. A twelve-week randomized controlled study of the cognitive-behavioral integrated psychological therapy program: positive effect on the social functioning of schizophrenic patients. Braz J Psychiatry. (2007) 29:140–7. doi: 10.1590/s1516-44462006005000030, PMID: 17650536

[40] Darmedru C Demily C Franck N. Preventing violence in schizophrenia with cognitive remediation. Encéphale. (2018) 44:158–67. doi: 10.1016/j.encep.2017.05.001, PMID: 28641817

[41] Sheitman BB Murray MG Snyder JA Silva S Goldman R Chakos M. Iq scores of treatment-resistant schizophrenia patients before and after the onset of the illness. Schizophr Res. (2000) 46:203–7. doi: 10.1016/s0920-9964(00)00034-7, PMID: 11120432

[42] Meier MH Caspi A Reichenberg A Keefe RS Fisher HL Harrington H. Neuropsychological decline in schizophrenia from the premorbid to the Postonset period: evidence from a population-representative longitudinal study. Am J Psychiatry. (2014) 171:91–101. doi: 10.1176/appi.ajp.2013.12111438, PMID: 24030246 PMC3947263

[43] Buchanan A Sint K Swanson J Rosenheck R. Correlates of future violence in people being treated for schizophrenia. Am J Psychiatry. (2019) 176:694–701. doi: 10.1176/appi.ajp.2019.18080909, PMID: 31014102

[44] Si TM. The expert consensus on psychiatric Management of Patients with agitation. Chin. J. Psychiatry. (2017) 06:401–10. doi: 10.3760/cma.j.issn.1006-7884.2017.06.002

[45] Yao J Tan SP Cui JF Chen N Fan HZ Xiao CL. Characteristics of aggressive behavior and related risk factors in inpatients with schizophrenia. Chin Ment Health J. (2018) 08:636–41. doi: 10.3969/j.issn.1000-6729.2018.08.003

[46] Yu FH Wang JX Ye XH Deng J Hang J Yang B. Ultrasound-based Radiomics nomogram: a potential biomarker to predict axillary lymph node metastasis in early-stage invasive breast cancer. Eur J Radiol. (2019) 119:108658. doi: 10.1016/j.ejrad.2019.108658, PMID: 31521878

[47] Santos TRM Melo JV Leite NC Salles GF Cardoso CRL. Usefulness of the vibration perception thresholds measurement as a diagnostic method for diabetic peripheral neuropathy: results from the Rio De Janeiro type 2 diabetes cohort study. J Diabetes Complicat. (2018) 32:770–6. doi: 10.1016/j.jdiacomp.2018.05.010, PMID: 29950276

